# Effects of prescribed aerobic exercise volume on physical activity and sedentary time in postmenopausal women: a randomized controlled trial

**DOI:** 10.1186/s12966-018-0659-3

**Published:** 2018-03-21

**Authors:** Jessica McNeil, Megan S. Farris, Yibing Ruan, Heather Merry, Brigid M. Lynch, Charles E. Matthews, Kerry S. Courneya, Christine M. Friedenreich

**Affiliations:** 1 Department of Cancer Epidemiology and Prevention Research, CancerControl Alberta, Alberta Health Services, Holy Cross Center, Room 514, Box ACB, 2210 2nd Street SW, Calgary, Alberta T2S 3C3 Canada; 2 Cancer Epidemiology and Intelligence Division, Cancer Council Victoria, Melbourne, VIC Australia; 3 Centre for Epidemiology and Biostatistics, Melbourne School of Population and Global Health, The University of Melbourne, Melbourne, VIC Australia; 4 Metabolic Epidemiology Branch, Division of Cancer Epidemiology and Genetics, National Cancer Institute, Bethesda, MD USA; 5 Faculty of Physical Education and Recreation, University of Alberta, Edmonton, AB Canada; 6 Departments of Oncology and Community Health Sciences, Cumming School of Medicine, University of Calgary, Calgary, AB Canada

**Keywords:** Exercise intervention, Exercise dose, Follow-up, Sedentary behavior

## Abstract

**Background:**

Physical activity has emerged as an important lifestyle factor for primary prevention of numerous diseases, including postmenopausal breast cancer. No study to date has assessed the acute and long-term effects of year-long aerobic exercise programs differing in prescribed exercise volume on physical activity and sedentary time in postmenopausal women. Therefore, we aimed to examine the effects of two moderate-vigorous intensity exercise doses on total, light and moderate-vigorous intensity physical activity times, and sedentary time in postmenopausal women during the year-long intervention and one year later.

**Methods:**

The Breast Cancer and Exercise Trial in Alberta (BETA) was a two-center, two-arm, 12-month randomized controlled trial that included 400 previously inactive postmenopausal women randomized to either 150 (MODERATE) or 300 (HIGH) minutes/week of aerobic exercise. Physical activity and sedentary time were assessed at baseline, 6- (intervention mid-point), 12- (prior to end of intervention) and 24-months (follow-up) with waist-mounted accelerometers (Actigraph GTX3®). Self-reported activity and sedentary time at baseline, 12- and 24-months was also assessed (Past Year Total Physical Activity Questionnaire and SIT-Q). Intention-to-treat analyses were conducted using linear mixed models and adjusted for baseline variables.

**Results:**

Both physical activity interventions led to increases in objective and subjective measures of total and moderate-vigorous intensity/recreational physical activity time, coupled with decreases in sedentary time, at 6- and 12-months compared to baseline. Additionally, greater increases in accelerometry-derived total physical activity time at 6- and 12-months, and self-reported recreational activity time at 12-months, compared to baseline were noted in the HIGH versus MODERATE groups. Decreases in total, light and moderate-vigorous intensity physical activity time, and an increase in sedentary time, in both groups were noted at 24-months compared to 12-months. A decrease in light intensity physical activity time in both groups at 24-months compared to baseline was also noted.

**Conclusion:**

These findings have important health implications, suggesting that total physical activity time can be increased with greater volumes of prescribed exercise, but that additional support and resources could be used to promote the maintenance of these high levels of aerobic exercise participation following study completion.

**Trial registration:**

clinicaltrials.gov identifier: NCT01435005 (BETA Trial). Registred September 15th 2011 (retrospectively registered).

**Electronic supplementary material:**

The online version of this article (10.1186/s12966-018-0659-3) contains supplementary material, which is available to authorized users.

## Background

Physical activity has emerged as an important lifestyle factor for primary prevention of numerous diseases including but not limited to: cardiovascular disease [1, 2], type 2 diabetes [3, 4] and certain types of cancers [5]. More recently, greater sedentary time has been identified as a risk factor for developing obesity and other adverse health outcomes [6, 7]. The menopausal transition in particular is associated with weight and fat mass gains [8, 9], which may partly result from decreased physical activity time [10, 11], hence leaving postmenopausal women at a heightened risk for developing adverse health outcomes.

The Canadian Society for Exercise Physiology recommends that adults should engage in at least 150 min of moderate-vigorous intensity aerobic physical activity/week to achieve health benefits [12]. However, findings from a Canadian nationally representative sample completed in 2007–2009 with objective measurements of physical activity time indicated that 85% of adults do not meet these guidelines [13]. Furthermore, this report noted a decline in total physical activity time coupled with an increase in sedentary time with age [13]. Therefore, the implementation of interventions and resources promoting increases in total physical activity time and lower sedentary time are needed, especially in older adults.

A modest number of intervention studies have examined the effects of an exercise and/or diet intervention on objectively-assessed physical activity time in postmenopausal women [14–20]. It still remains uncertain whether higher volumes of prescribed exercise (300 min/week) would lead to greater increases in physical activity time and better maintenance of this increase over the long term. Two studies have used pedometers as an objective measurement of physical activity time [19, 20]. The Women On the Move through Activity and Nutrition (WOMAN) study found that pedometer steps were significantly higher in the lifestyle intervention compared to the health education group [19]. The Dose Response to Exercise in Women (DREW) Trial found a statistically significant linear trend between the lowest and highest volume of exercise prescribed using pedometer data [20]. Two more recent studies used accelerometer devices [14, 18]. Specifically, The Diet, Exercise, Metablism, and Obesity in Older Women (DEMO) Trial randomized 36 postmenopausal women to caloric restriction interventions combined with either a moderate- or vigorous-intensity aerobic exercise component [18]. This study found that participants had greater physical activity time on days when they did not perform center-based/supervised exercises, and that this difference was especially greater for participants randomized to the vigorous-intensity aerobic exercise group. Additionally, in the Sex Hormones and Physical Exercise (SHAPE)-2 study, women randomized to the exercise intervention were more physically active and less sedentary at follow-up (12-months post intervention) [14].

The Breast Cancer and Exercise Trial in Alberta (BETA) assessed the dose-response effects of a year-long prescribed aerobic exercise intervention on biomarkers related to breast cancer risk in postmenopausal women [21]. We collected objective measurements of physical activity and sedentary time before, during and just prior to the end of the intervention (baseline, 6- and 12-months), as well as at follow-up (24-months). We also collected self-reported, domain-specific measures of physical activity and sedentary time at baseline, 12- and 24-months. The objective of the present analysis was to examine the effects of prescribed aerobic exercise volume on physical activity and sedentary time at 6-, 12- and 24-months.

## Methods

### Setting and participants

The design and methods for BETA are described in detail elsewhere [21–23]. This two-center, two-arm randomized controlled exercise intervention trial and follow-up assessments were conducted in Calgary and Edmonton (Alberta, Canada) between June 2010 and June 2014. A total of 400 women were randomized to the MODERATE (150 min of exercise/week) or HIGH (300 min of exercise/week) volumes of aerobic exercise interventions. Eligibility criteria included: age 50–74 years, postmenopausal, no previous cancer diagnosis, inactive (< 90 min/week of exercise or if between 90 and 120 min/week, a VO_2peak_ < 34 ml/kg/min as measured by a submaximal fitness test), a body mass index (BMI) between 22 and 40 kg/m^2^, non-smoker, able to do unrestricted exercise as assessed by physician screening, and not planning to undertake a weight loss or dietary program. The study protocol was approved by the Alberta Cancer Research Ethics Committee, the Conjoint Health Research Ethics Board of the University of Calgary and the Health Research Ethics Board of the University of Alberta. Informed consent was provided by all participants prior to study participation. This study followed the CONSORT guidelines for reporting [24].

### Exercise interventions

All participants were asked to exercise five days/week accomplishing 65–75% of heart rate reserve for either 30 min (MODERATE) or 60 min (HIGH)/session. All participants wore heart rate monitors to ensure that the exercise was completed within the prescribed target heart rate zones. Exercise sessions were supervised by certified exercise trainers at fitness facilities in Calgary and Edmonton on at least three days/week. Exercise on the other two days/week could be unsupervised and completed at a location of the participants choosing. Participants could choose to exercise at the fitness facilities on five days/week. Initially, exercise volume increased gradually over a 12-week ramp-up period [21, 22, 25]. Exercise adherence was monitored with weekly exercise logs completed by the exercise trainers. The exercise trainers also recorded information collected by the heart rate monitors (exercise time, continuous heart rate, time spent in the pre-determined heart rate zones and the type of aerobic activities completed) during supervised and unsupervised exercise sessions into a database that was maintained at the recreational facilities in Edmonton and Calgary during the year-long intervention. Participants were aware that all outcome assessments would be repeated at the 24-month time-point. However, physical activity time was not monitored, nor did participants receive an exercise prescription or had access to the training facilities and exercise trainers during the 12- to 24-month period.

### Outcome measures

All outcomes were assessed at baseline, intervention mid-point (6-months), just prior to the end of intervention (12-months) and at follow-up (24-months). Total, moderate-vigorous and light intensity physical activity times, and sedentary time were objectively-assessed with the ActiGraph® GT3X+ device (ActiGraph LLC, Pensacola, Florida, USA). Participants were asked to wear the activity monitor around their waist during all waking hours (i.e. monitors were removed during sleep), except for water-based activities, for seven consecutive days at each time-point. A daily activity monitor log was completed by each participant to record the time when the accelerometer was worn and the types of activities done during any “non-wear” time throughout the day. Accelerometry data were collected from the ActiGraph® GT3X+ device at a sampling rate of 80 Hz and were aggregated to 60-s epoch files for analysis by the ActiLife® software (v6.10.2). For accelerometer data to be deemed valid at each time-point, at least four days of 10 h of wear time/day were needed. This amount of valid data was verified first with graphical displays computed by the ActiLife® software and the participants’ daily activity monitor logs at data collection and then through data processing. For cases of invalid data at the collection phase, study staff asked participants to wear the device for another seven consecutive days. We used the Actigraph® Vertical Axis (VT) calculations [26, 27] to derive physical activity and sedentary time outcomes from the accelerometry-measured activity counts. First, total physical activity time (Metabolic Equivalents of Task, METs) was calculated with the following regression equation proposed by Freedson et al. [27]: METs/min = 1.439008 + (0.000795 x vertical axis counts/min). These values per minute were then summed and divided by 60 to obtain total physical activity time values in MET-hours/day. Second, the following cut-points were used to define physical activity time according to intensity and sedentary time for the VT calculations: < 100 counts per minute (sedentary), 100–760 counts per minute (light intensity) and > 760 counts per minute (moderate-vigorous intensity). We applied the Choi algorithm to remove non-wear time [28]. To account for potentially influential non-wear time during activities captured in the activity monitor logs filled out by participants, we first assigned a specific MET value to the activity reported in the monitor logs according to the values presented in the Compendium of Physical Activities [29]. However, these values were not compatible with the accelerometry-collected data. Therefore, we attempted to apply a mean imputation value of three METs for every hour of non-wear activity that was documented [30], independently of the reported activity that was performed during non-wear time. This mean imputation was only applied when an activity (e.g. cycling, jogging) was reported by the participants in the activity logs during non-wear time. No statistically significant differences were noted between mean imputed and non-imputed total physical activity time (results not shown). Hence, we decided not to include imputed non-wear activity values into our estimates of physical activity time.

Self-reported activity and sedentary time were assessed with the Past Year Total Physical Activity Questionnaire (PYTPAQ) [31] and the SIT-Q [32] at baseline, 12- and 24-months. These questionnaires assessed usual activity and sedentary time within the last 12 months. All activities reported on the PYTPAQ were converted into MET-hours/week using the Compendium of Physical Activities [29]. The derived variables from the PYTPAQ included: total, occupational, recreational, household and transportation activities. The derived variables from the SIT-Q included: total, occupational and leisure sedentary time, all reported as hours/day.

### Statistical analyses

The sample size for BETA was based on the primary endpoint of adiposity [22]. Sample size calculations estimated an initial sample size of 165 participants/group at 12-months. We expected a < 15% loss at 24-months, suggesting that at least 140 participants would be included in the 24-month analyses [23]. For the present analyses, it is estimated that the final sample sizes of 140–170 participants per group with a pre-determined power of 0.80 and a two-tailed α = 0.05 provided an effect size of Cohen’s *d* = 0.30–0.34 (small-medium effect) to detect differences in total physical activity and sedentary time outcomes between groups at 6-, 12- and 24-months compared to baseline.

Differences in baseline participant characteristics between the MODERATE and HIGH groups were assessed with a two-sample t-test for continuous variables or a χ^2^ test for categorical variables. An intention-to-treat analysis that included all participants with complete accelerometry data at baseline, 6-, 12- and/or 24-months regardless of protocol adherence was used. This same approach was used for the PYTPAQ and SIT-Q outcome data at baseline, 12- and 24-months. Generalized linear models estimated least-squares mean differences (LSMD) in the change of physical activity and sedentary time outcomes between the MODERATE and HIGH groups for each time-point comparison. These models were a priori adjusted for baseline values of age (years), body mass index (BMI; kg/m^2^) and VO_2peak_ (submaximal cardiorespiratory test; ml/kg/min), as well as differences in wear time (hours/day) between time-point comparisons (for accelerometry data only). Full-time employment status (work ≥ 35 h/week or < 35 h/week) was also added as a covariate in the SIT-Q data analysis. Potential effect modification for accelerometry-derived total physical activity and sedentary time in the baseline to 12 months and baseline to 24 months analyses were assessed for the following variables based on their scientific plausibility and support in previous research [33–35]: age (years), marital status (married/common law or other), study site (Calgary or Edmonton), education (≤ high school or ≥ post-secondary school), ethnicity (Caucasian or other), baseline VO_2peak_ (ml/kg/min) and baseline BMI (kg/m^2^). Since no evidence of effect modification was noted (results not shown), stratified analyses were not conducted. Potential confounding was assessed on the remaining variables not adjusted for a priori using backwards elimination. All analyses were performed using SAS (SAS 9.2 for Linux, SAS Institute Inc.) [36]. Statistical significance was set at *α* <  0.05.

## Results

**Fig. 1:**
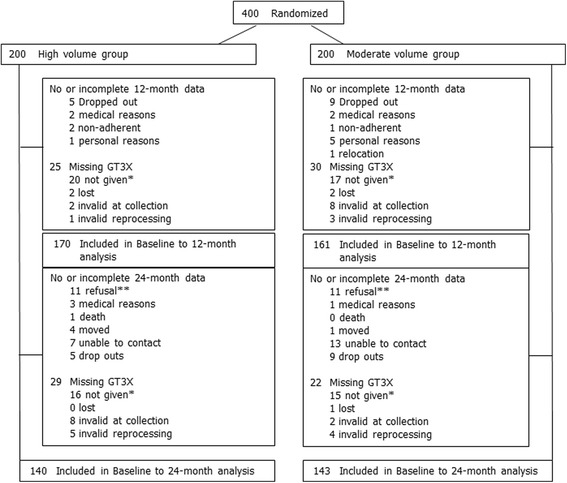
CONSORT diagram; the flow of participants through the BETA study, Alberta, Canada, 2010–2014. *Accelerometers were not given to participants who had low adherence throughout the intervention or refused to wear the accelerometer a second time following invalid data (e.g. device malfunction, did not wear the accelerometer for at least four days for 10 or more hours/day). **Participants who refused to wear the accelerometer and/or wanted only to complete the study questionnaires at the 24-month time-point

The number of referred/invited and eligible participants, as well as reasons for exclusion from randomization, were previously reported [22]. Of the 400 participants randomized at baseline, 331 and 283 participants had complete and valid accelerometry data at baseline to 12-months and baseline to 24-months, respectively (Fig. 1; Study Flow Chart). A total of 321 and 272 participants had complete and valid accelerometry data at baseline to 6-months and 12- to 24-months, respectively. Baseline participant characteristics for those who had valid and complete accelerometry data at baseline to 12-months (*n* = 331) are presented in Table 1. There were no group differences in accelerometer wear time at baseline (15.0 ± 1.1 versus 15.1 ± 1.1 h/day; *P* = 0.7), 6-months (14.7 ± 1.1 versus 14.9 ± 1.1 h/day; *P* = 0.2), 12-months (14.9 ± 1.1 versus 14.9 ± 1.1 h/day; *P* = 0.6) and 24-months (14.9 ± 1.1 versus 14.8 ± 1.1 h/day; *P* = 0.8).

**Table 1:** Baseline covariates for the participants randomized to the HIGH and MODERATE exercise groups in the Breast Cancer and Exercise Trial in Alberta (BETA) (*n* = 331)

Characteristics	HIGH group (300 min/week) *n* = 170	MODERATE group (150 min/week) *n* = 161	*P*-value
Age (years); *mean ± SD*	59 ± 5	59 ± 5	0.56
Baseline BMI (kg); *mean ± SD*	29 ± 4	29 ± 4	0.35
Baseline VO_2peak_ (ml kg^− 1^ min^− 1^); *mean ± SD*	27 ± 5	27 ± 5	0.97
Study site			
Calgary; *n (%)*	125 (74)	120 (75)	0.84
Edmonton; *n (%)*	45 (26)	41 (25)	
Marital status			
Married or common law; *n (%)*	120 (71)	112 (70)	0.84
Other; *n (%)*	50 (29)	49 (30)	
Education			
≤ High school; *n (%)*	35 (21)	37 (23)	0.60
≥ Post-secondary school; *n (%)*	135 (79)	124 (77)	
Ethnicity			
Caucasian; *n (%)*	145 (85)	147 (91)	0.09
Other; *n (%)*	25 (15)	14 (9)	

*BMI* Body mass index, *SD* Standard deviation, *VO2peak* Maximal oxygen uptake

Data on exercise adherence obtained from the exercise logs maintained by the BETA Exercise Trainers are presented elsewhere [37–39]. There was no statistical difference in the number of completed supervised (2.2 ± 0.7 versus 2.1 ± 0.7 sessions/week; *P* = 0.4) and unsupervised (1.6 ± 0.6 versus 1.5 ± 0.6 sessions/week; *P* = 0.8) sessions between groups. For participants included in the accelerometry analyses, the HIGH group recorded greater exercise time during the intervention (two-sample *t*-test; *P* <  0.0001), with 12-month median (quartiles 1, 3) adherence values of 239 (193, 264) and 132 (115, 139) minutes/week. The median adherence rates for the HIGH and MODERATE groups for weeks 13–52 (full exercise prescription following the ramp-up period) were 262 (203, 292) and 140 (121, 150) minutes/week, respectively (two-sample *t*-test; *P* <  0.0001).

Statistically significant increases in total and moderate-vigorous intensity physical activity time, and a decrease in sedentary time, were noted in both groups at 12-months compared to baseline (Table 2). Similar results were noted for changes in accelerometry-derived outcomes between baseline and 6-months (Additional file 1). However, only the increase in total physical activity time was greater in the HIGH versus MODERATE group at 6-months, and borderline significant at 12-months, compared to baseline. A statistically significant increase in self-reported total and recreational activity time, and a decrease in leisure sedentary time, were noted in both groups at 12-months compared to baseline (Additional file 2). This increase in self-reported recreational activity time was also greater in the HIGH vs. MODERATE group between these time points.

**Table 2:** Changes in accelerometry-derived physical activity and sedentary time variables (baseline to 12-months) between HIGH and MODERATE groups in BETA, Alberta, Canada, 2010–2014

Outcome measure ^a^	Baseline M (SD)	12-months M (SD)	LS Mean Change M (95% CI)	*P* value ^b^	LS Group Difference M (95% CI)	*P* value ^d^
Total physical activity time (MET-h/d)						
HIGH	25.2 (2.2)	26.3 (2.6)	1.03 (0.82, 1.23)	< 0.001	0.29 (−0.0002, 0.59)	0.05
MODERATE	25.2 (2.1)	25.8 (2.4)	0.73 (0.52, 0.95)	< 0.001		
Total physical activity time (MET-h/wk)						
HIGH	176.4 (15.4)	184.1 (18.2)	7.21 (5.74, 8.61)		2.03 (−0.014, 4.13)	
MODERATE	176.4 (14.7)	180.6 (16.8)	5.11 (5.74, 8.61)			
Moderate-vigorous physical activity time (h/d)						
HIGH	2.0 (0.8)	2.3 (0.8)	0.34 (0.25, 0.44)	< 0.001	0.12 (−0.03, 0.26)	0.11
MODERATE	2.0 (0.8)	2.2 (0.8)	0.23 (0.13, 0.33)	< 0.001		
Moderate-vigorous physical activity time (h/wk)						
HIGH	14.0 (5.6)	16.1 (5.6)	2.38 (1.75, 3.08)		0.84 (− 0.21, 1.82)	
MODERATE	14.0 (5.6)	15.4 (5.6)	1.61 (0.91, 2.31)			
Light activity time (h/d)						
HIGH	4.3 (1.0)	4.3 (0.9)	0.01 (− 0.09, 0.11)	0.88	0.01 (−0.13, 0.15)	0.94
MODERATE	4.2 (0.8)	4.2 (0.9)	0.002 (−0.10, 0.10)	0.97		
Light activity time (h/wk)						
HIGH	30.1 (7.0)	30.1 (6.3)	0.07(−0.63, 0.77)		0.07 (−0.91, 1.05)	
MODERATE	29.4 (5.6)	29.4 (6.3)	0.01 (− 0.70, 0.70)			
Sedentary time (h/d)						
HIGH	8.7 (1.6)	8.3 (1.4)	−0.48 (− 0.64, − 0.32)	< 0.001	− 0.13 (− 0.36, 0.11)	0.28
MODERATE	8.9 (1.6)	8.5 (1.6)	−0.35 (− 0.52, − 0.18)	< 0.001		
Sedentary time (h/wk)						
HIGH	60.9 (11.2)	58.1 (9.8)	−3.36 (−4.48, −2.24)		−0.91 (−2.52, 0.77)	
MODERATE	62.3 (11.2)	59.5 (11.2)	−2.45 (− 3.64, −1.26)			

*CI* Confidence interval, *d* Day, *h* Hours, *LS* Least-squares, *M* Mean, *MET* Metabolic equivalent of task, *SD* Standard deviation, *wk* Week

^a^n = 170 and 161 for the HIGH and MODERATE groups, respectively

^b^*P* value for the test of significance for the null hypothesis that the LS mean difference across time equals 0

^c^Least-square group mean of the High and Moderate exercise groups and their within- and between-group differences were estimated from general linear models specified as: physical activity and sedentary time changes from baseline to 12-months = β0 + β1 (intervention group) + β2 (baseline outcome value) + β3 (age) + β4 (study site) + β5 (baseline BMI) + β6 (baseline VO2peak) + β7 (difference in accelerometer wear time between time-points)

^d^*P* value for the test of significance for the null hypothesis that the LS mean difference between the two intervention groups equals 0

A statistically significant decrease in total, light and moderate-vigorous intensity physical activity time, and an increase in sedentary time, in both groups were noted at 24-months compared to 12-months (Table 3). However, no differences in accelerometry-derived variables were noted between groups between these time points. A statistically significant decrease in self-reported total and recreational activity were noted in both groups at 24-months compared to 12-months (Additional file 3). Furthermore, this decrease in self-reported recreational activity was greater in the HIGH versus MODERATE group between these time points.

**Table 3:** Changes in accelerometry-derived physical activity and sedentary time variables (12- to 24-months) between HIGH and MODERATE groups in BETA, Alberta, Canada, 2010–2014

Outcome measure ^a^	12-months M (SD)	24-months M (SD)	LS Mean Change ^b^ M (95% CI)	*P* value ^c^	LS Group Difference ^b^ M (95% CI)	*P* value ^d^
Total physical activity time (MET-h/d)						
HIGH	26.5 (2.6)	25.2 (2.5)	−1.29 (− 1.48, − 0.88)	< .001	− 0.22 (− 0.49, 0.05)	0.11
MODERATE	26.2 (2.2)	25.1 (2.4)	− 1.07 (− 1.26, − 0.88)	< .001		
Total physical activity time (MET-h/wk)						
HIGH	185.5 (18.2)	176.4 (17.5)	−9.03 (− 10.36, −6.16)		−1.54 (−3.43, 0.35)	
MODERATE	183.4 (15.4)	175.7 (16.8)	−7.49 (−8.82, −6.16)			
Moderate-vigorous physical activity time (h/d)						
HIGH	2.3 (0.8)	1.9 (0.8)	−0.41 (− 0.50, − 0.31)	< .001	− 0.05 (− 0.18, 0.09)	0.50
MODERATE	2.2 (0.8)	1.9 (0.8)	− 0.36 (− 0.46, − 0.26)	< .001		
Moderate-vigorous physical activity time (h/wk)						
HIGH	16.1 (5.6)	13.3 (5.6)	−2.87 (−3.50, − 2.17)		− 0.35 (− 1.26, 0.63)	
MODERATE	15.4 (5.6)	13.3 (5.6)	−2.52 (−3.22, − 1.82)			
Light activity time (h/d)						
HIGH	4.4 (0.9)	4.2 (0.9)	−0.17 (− 0.28, − 0.06)	0.002	0.02 (− 0.14, 0.17)	0.82
MODERATE	4.3 (0.9)	4.1 (0.9)	−0.19 (− 0.30, − 0.08)	< .001		
Light activity time (h/wk)						
HIGH	30.8 (6.3)	29.4 (6.3)	−1.19 (− 1.96, − 0.42)		0.14 (− 0.98, 1.19)	
MODERATE	30.1 (6.3)	28.7 (6.3)	−1.33 (−2.10, − 0.56)			
Sedentary time (h/d)						
HIGH	8.3 (1.4)	8.8 (1.6)	0.44 (0.27, 0.60)	< .001	0.04 (−0.19, 0.27)	0.75
MODERATE	8.5 (1.4)	8.9 (1.5)	0.40 (0.23, 0.56)	< .001		
Sedentary time (h/wk)						
HIGH	58.1 (9.8)	61.6 (11.2)	3.08 (1.89, 4.20)		0.28 (−1.33, 1.89)	
MODERATE	59.5 (9.8)	62.3 (10.5)	2.80 (1.61, 3.92)			

*CI* Confidence interval, *d* Day, *h* Hours, *LS* Least-squares, *M* Mean, *MET* Metabolic equivalent of task, *SD* Standard deviation, *wk* Week

^a^*n* = 138 and 134 for the HIGH and MODERATE groups, respectively

^b^Least-square group mean of the High and Moderate exercise groups and their within- and between-group differences were estimated from general linear models specified as: physical activity and sedentary time changes from 12- to 24-months = β0 + β1 (intervention group) + β2 (12-month outcome value) + β3 (age) + β4 (study site) + β5 (baseline BMI) + β6 (baseline VO2peak) + β7 (difference in wear time between time-points)

^c^*P* value for the test of significance for the null hypothesis that the LS mean difference across time equals 0

^d^*P* value for the test of significance for the null hypothesis that the LS mean difference between the two intervention groups equals 0

A statistically significant decrease in total physical activity time in the MODERATE group only and light intensity activity time in both groups at 24-months compared to baseline was observed (Table 4). No between-group differences in accelerometry-derived outcomes were noted between these time points. A statistically significant increase in self-reported total and recreational activity, and a decrease in leisure sedentary time, in both groups at 24-months compared to baseline was noted (Additional file 4). A decrease in self-reported total sedentary time at 24-months compared to baseline was also noted in the HIGH group only. There were no between-group differences in self-reported activity and sedentary time measurements between these time points.

**Table 4:** Changes in accelerometry-derived physical activity and sedentary time variables (baseline to 24-months) between HIGH and MODERATE groups in BETA, Alberta, Canada, 2010–2014

Outcome measure ^a^	Baseline M (SD)	24-months M (SD)	LS Mean Change ^b^ M (95% CI)	*P* value ^c^	LS Group Difference ^b^ M (95% CI)	*P* value ^d^
Total physical activity time (MET-h/d)						
HIGH	25.3 (2.2)	25.2 (2.5)	−0.19 (− 0.40, 0.03)	0.09	0.04 (− 0.26, 0.35)	0.80
MODERATE	25.3 (2.2)	25.0 (2.4)	−0.23 (− 0.44, − 0.01)	0.04		
Total physical activity time (MET-h/wk)						
HIGH	177.1 (15.4)	176.4 (17.5)	−1.33 (−2.80, 0.21)		0.28 (−1.82, 2.45)	
MODERATE	177.1 (15.4)	175.0 (16.8)	−1.61 (−3.08, −0.07)			
Moderate-vigorous physical activity time (h/d)						
HIGH	1.9 (0.8)	1.9 (0.8)	−0.05 (− 0.16, 0.06)	0.38	0.03 (− 0.13, 0.19)	0.72
MODERATE	1.9 (0.9)	1.9 (0.8)	−0.08 (− 0.19, 0.03)	0.16		
Moderate-vigorous physical activity time (h/wk)						
HIGH	13.3 (5.6)	13.3 (5.6)	−0.35 (−1.12, 0.42)		0.21 (−0.91, 1.33)	
MODERATE	13.3 (6.3)	13.3 (5.6)	−0.56 (−1.33, 0.21)			
Light activity time (h/d)						
HIGH	4.4 (1.0)	4.2 (0.9)	−0.17 (−0.28, − 0.05)	0.004	−0.02 (− 0.18, 0.13)	0.78
MODERATE	4.3 (0.8)	4.1 (0.9)	−0.14 (− 0.25, − 0.03)	0.01		
Light activity time (h/wk)						
HIGH	30.8 (7.0)	29.4 (6.3)	−1.19 (−1.96, −0.35)		−0.14 (− 1.26, 0.91)	
MODERATE	30.1 (5.6)	28.7 (6.3)	−0.98 (−1.75, − 0.21)			
Sedentary time (h/d)						
HIGH	8.8 (1.6)	8.8 (1.5)	−0.07 (− 0.24, 0.10)	0.43	0.02 (− 0.27, 0.23)	0.88
MODERATE	9.0 (1.5)	8.8 (1.5)	−0.05 (− 0.22, 0.12)	0.56		
Sedentary time (h/wk)						
HIGH	61.6 (11.2)	61.6 (10.5)	−0.49 (−1.68, 0.70)		0.14 (− 1.89, 1.61)	
MODERATE	63.0 (10.5)	61.6 (10.5)	−0.35 (−1.54, 0.84)			

*CI* Confidence interval, *d* Day, *h* Hours, *LS* Least-squares, *M* Mean, *MET* Metabolic equivalent of task, *SD* Standard deviation, *wk* Week

^a^*n* = 140 and 143 for the HIGH and MODERATE groups, respectively

^b^Least-square group mean of the High and Moderate exercise groups and their within- and between-group differences were estimated from general linear models specified as: physical activity and sedentary time changes from baseline to 24-months = β0 + β1 (intervention group) + β2 (baseline outcome value) + β3 (age) + β4 (study site) + β5 (baseline BMI) + β6 (baseline VO2peak) + β7 (difference in wear time between time-points)

^c^*P* value for the test of significance for the null hypothesis that the LS mean difference across time equals 0

^d^*P* value for the test of significance for the null hypothesis that the LS mean difference between the two intervention groups equals 0

## Discussion

To our knowledge, this is the first study to assess the effects of different volumes of prescribed aerobic exercise on total physical activity and sedentary time in postmenopausal women during a 12-month intervention and at 24-month follow-up. We noted increases in objective and subjective measures of total and moderate-vigorous intensity/recreational physical activity time, coupled with decreases in sedentary time, in response to both volumes of prescribed physical activity at 6- and 12-months. These results are consistent with findings from a six-week randomized controlled trial by Gomersall et al. that prescribed 300 min/week versus 150 min/week of aerobic physical activity versus no additional activity (control) in previously inactive adults [40]. That study reported statistically significant increases in total physical activity time in both exercise interventions compared to control [40]. Additionally, our results confirmed that greater volumes of prescribed exercise led to greater increases in total physical activity time at 6- and 12-months compared to baseline. The smaller difference in total physical activity time change observed between groups at 12- compared to 6-months (i.e. difference of 0.46 versus 0.29 MET-hours/day between groups at 6- and 12-months) may be partly explained by slight differences in the completion rates of prescribed exercise sessions following the ramp-up period between groups (91% and 84% in the MODERATE and HIGH exercise groups) and/or greater improvements in exercise economy/efficiency over time with higher training volumes [41]. Data on self-reported activity that we obtained via the PYTPAQ corroborate these findings, indicating that recreational activity was significantly greater at 12-months compared to baseline in the HIGH versus MODERATE exercise groups. As previously reported [22], mean reductions in total body fat, abdominal fat, BMI, waist circumference and waist to hip ratio were greater in the HIGH vs. MODERATE groups at 12-months compared to baseline, with the greatest reductions in adiposity occurring in participants reporting more than 250 min/week of exercise. It is therefore suggested that greater volumes of aerobic exercise led to greater decreases in adiposity measures in these participants.

Decreases in total, light and moderate-vigorous intensity physical activity time, and an increase in sedentary time, in both groups were noted at 24-months compared to 12-months. Additionally, decreases in self-reported total and recreational activity were observed in both groups at 24-months compared to 12-months, with significantly greater reductions in self-reported recreational activity in the HIGH versus MODERATE groups at 24-months compared to 12-months. A decrease in light intensity physical activity time in both groups and a decrease in total physical activity time in the MODERATE group only were also noted at 24-months compared to baseline. Our recent paper examining the changes in adiposity measurements in BETA at follow-up reported increases in total body fat between 12 and 24 months in both groups [23], which may be partially explained by this decrease in total physical activity time following study completion. Gomersall et al. [40] reported similar findings, indicating that total physical activity time returned to baseline levels in all groups (300 min/week versus 150 min/week versus control) six months following the end of the six-week intervention. Conversely, other studies reported an increase in moderate-vigorous intensity physical activity time and/or lower sedentary time at follow-up versus baseline [14, 42]. A meta-analysis indicated that the degree of energy compensation (i.e. amount of weight loss below what is expected for the amount of exercise energy expenditure) is highly variable for exercise interventions of short durations (< 20 weeks), but approaches 100% (i.e. no changes in body weight, despite increased exercise participation) in exercise interventions > 40 weeks in duration [43]. It is possible that delayed compensatory responses in total physical activity time and/or sedentary time may be greater in BETA as a result of the length of the intervention and follow-up periods.

It is very likely that decreases in total physical activity time and/or increases in sedentary time are expected to occur following the end of an exercise intervention (i.e. the changes in our outcome measures reflect the removal of the intervention rather than the maintenance of an intervention), thus highlighting the challenges of implementing these types of exercise interventions or recommendations into the real world. Indeed, one study speculated that it may be difficult for participants in a structured exercise intervention group to find new ways/settings to maintain their physical activity routine following study completion, compared to those randomized to a home-based physical activity program [44]. Recent studies have tried to implement strategies to prevent reversions in total physical activity time following the completion of exercise interventions. More specifically, a two-arm intervention by Niklas et al. [45] investigated the effects of adding a self-monitoring intervention with accelerometry to a traditional diet and exercise trial, versus diet and exercise alone, on total physical activity time and anthropometrics during a five-month weight maintenance period in older overweight adults. The authors reported a positive trend towards greater light intensity activity time in the group with the added self-monitoring intervention compared to diet and exercise alone. Cadmus-Bertram et al. [46, 47] also recently reported an increase in moderate-vigorous intensity physical activity time following a 16-week self-monitoring exercise intervention with a Fitbit® activity tracker in postmenopausal women. Additionally, 96% of participants found the Fitbit® activity tracker to be “somewhat or very helpful” for increasing total physical activity time. Therefore, the addition of self-monitoring tools in conjunction with a structured exercise program may contribute to maintaining increased total physical activity and reduced sedentary time following study completion. Future studies evaluating the use of self-monitoring tools to promote the maintainance of increased physical activity and reduced sedentary time following an exercise intervention are needed to corroborate the abovementioned findings.

Our results are mostly generalizable to healthy, postmenopausal women. Strengths of BETA include the implementation of a year-long exercise intervention including supervised exercise sessions, a high adherence rate in both exercise groups, a large sample size and the inclusion of both objective and subjective assessments of physical activity and sedentary time during the intervention, at study completion and follow-up. Our limitations include the large number of analyses conducted, which may increase the chances of spurious findings. Additionally, there were missing accelerometry data in all analyses. The most common reasons for missing accelerometer data included refusal to wear the device and lack of adherence to the trial, which may be evidence of selection bias, since women who refused to wear the devices may be more inactive than those who did not. However, when comparing participants with valid accelerometry data at baseline and 12-months (*n* = 331) versus participants without valid accelerometry data (*n* = 69), age, BMI, VO_2peak_ and measures of physical activity and sedentary time did not differ between completers and non-completers, with the exception of light intensity activity time (*P* = 0.01). Thus, the magnitude of selection bias is minimal and may not influence our overall results.

These findings suggest that total physical activity time can be increased with greater volumes of prescribed exercise. However, physical activity and sedentary time returned to baseline levels following study completion, thus highlighting the challenges in implementing these types of exercise interventions or recommendations into the real world. Additional support and resources (e.g. use of self-monitoring activity trackers) may be beneficial to promote the maintenance of increased physical activity and reduced sedentary time over the long-term following study completion. Future studies should also include qualitative and/or quantitative assessments of common barriers to maintaining achieved increases in physical activity participation and reduced sedentary time following an exercise intervention.

## Additional files

Additional file 1 Changes in accelerometry-derived physical activity and sedentary time variables (baseline to 6-months) between HIGH and MODERATE groups in BETA, Alberta, Canada, 2010–2014. (DOCX 18 kb)

Additional file 2 Changes in self-reported physical activity and sedentary time variables (baseline to 12-months) between HIGH and MODERATE groups in BETA, Alberta, Canada, 2010–2014. (DOCX 23 kb)

Additional file 3 Changes in self-reported physical activity and sedentary time variables (12- to 24-months) between HIGH and MODERATE groups in BETA, Alberta, Canada, 2010–2014. (DOCX 22 kb)

Additional file 4 Changes in self-reported physical activity and sedentary time variables (baseline to 24-months) between HIGH and MODERATE groups in BETA, Alberta, Canada, 2010–2014. (DOCX 23 kb)

## Abbreviations

BETA: Breast Cancer and Exercise Trial in Alberta
BMI: Body Mass Index
CI: Confidence Interval
LSMD: Least-Squares Mean Difference
MET: Metabolic Equivalent of Task
PYTPAQ: Past Year Total Physical Activity Questionnaire
VT: Vertical Axis
