# Supplementary material for: Effects of prescribed aerobic exercise volume on physical activity and sedentary time in postmenopausal women: a randomized controlled trial
Source: Int J Behav Nutr Phys Act. 2018 Mar 21;15:27. doi: 10.1186/s12966-018-0659-3 (PMC5863375; doi:10.1186/s12966-018-0659-3)
Supplement: Supplementary file 1 — Changes in accelerometry-derived physical activity and sedentary time variables (baseline to 6-months) between HIGH and MODERATE groups in BETA, Alberta, Canada, 2010–2014. (DOCX 18 kb) [file 12966_2018_659_MOESM1_ESM.docx]

**Additional file 1.** Changes in accelerometry-derived physical activity and sedentary time variables (baseline to 6-months) between HIGH and MODERATE groups in BETA, Alberta, Canada, 2010-2014.

| **Outcome measure ^a^** | **Baseline**  M (SD) | **6-months**  M (SD) | **LS Mean Change ^b^**  M (95% CI) | *P* value ^c^ | **LS Group Difference ^b^**  M (95% CI) | *P* value ^d^ |
| --- | --- | --- | --- | --- | --- | --- |
| Total physical activity time (MET-h/d)  HIGH  MODERATE  Total physical activity time (MET-h/wk)  HIGH  MODERATE | 25.0 (2.2)  25.2 (2.1)  175.0 (15.4)  176.4 (14.7) | 26.2 (2.7)  26.0 (2.2)  183.4 (18.9)  182.0 (15.4) | 1.08 (0.86, 1.30)  0.61 (0.39, 0.84)  7.56 (6.02, 9.10)  4.27 (2.73, 5.88) | < 0.001  < 0.001 | 0.46 (0.16, 0.76)  3.22 (1.12, 5.32) | 0.002 |
| Moderate-vigorous physical activity time (h/d)  HIGH  MODERATE  Moderate-vigorous physical activity time (h/wk)  HIGH  MODERATE | 1.9 (0.8)  2.0 (0.9)  13.3 (5.6)  14.0 (6.3) | 2.3 (0.8)  2.3 (0.8)  16.1 (5.6)  16.1 (5.6) | 0.39 (0.29, 0.49)  0.29 (0.19, 0.40)  2.73 (2.03, 3.43)  2.03 (1.33, 2.80) | < 0.001  < 0.001 | 0.10 (-0.04, 0.24)  0.7 (-0.28, 1.68) | 0.17 |
| Light activity time (h/d)  HIGH  MODERATE  Light activity time (h/wk)  HIGH  MODERATE | 4.3 (1.0)  4.2 (0.8)  30.1 (7.0)  29.4 (5.6) | 4.2 (0.9)  4.3 (0.8)  29.4 (6.3)  30.1 (5.6) | -0.05 (-0.15, 0.06)  0.06 (-0.04, 0.17)  -0.35 (-1.05, 0.42)  0.42 (-0.28, 1.19) | 0.36  0.24 | -0.11 (-0.25, 0.03)  -0.77 (-1.75, 0.21) | 0.12 |
| Sedentary time (h/d)  HIGH  MODERATE  Sedentary time (h/wk)  HIGH  MODERATE | 8.7 (1.5)  8.9 (1.6)  60.9 (10.5)  62.3 (11.2) | 8.1 (1.4)  8.4 (1.6)  56.7 (9.8)  58.8 (11.2) | -0.56 (-0.73, -0.39)  -0.52 (-0.69, -0.34)  -3.92 (-5.11, -2.73)  -3.64 (-4.83, -2.38) | < 0.001  < 0.001 | -0.04 (-0.28, 0.19)  -0.28 (-1.96, 1.33) | 0.72 |

**Note:** CI, confidence interval; d, day; h, hours; LS, least-squares; M, mean; MET, metabolic equivalent of task; SD, standard deviation; wk, week.

^a^ n = 165 and 156 for the HIGH and MODERATE groups, respectively.

^b^ Least-square group mean of the High and Moderate exercise groups and their within- and between-group differences were estimated from general linear models specified as: physical activity and sedentary time changes from baseline to 6-months = β0 + β1 (intervention group) + β2 (baseline outcome value) + β3 (age) + β4 (study site) + β5 (baseline BMI) + β6 (baseline VO2peak) + β7 (difference in wear time between time-points).

^c^ *P* value for the test of significance for the null hypothesis that the LS mean difference across time equals 0.

^d^ *P* value for the test of significance for the null hypothesis that the LS mean difference between the two intervention groups equals 0.
