# Supplementary material for: Effects of prescribed aerobic exercise volume on physical activity and sedentary time in postmenopausal women: a randomized controlled trial
Source: Int J Behav Nutr Phys Act. 2018 Mar 21;15:27. doi: 10.1186/s12966-018-0659-3 (PMC5863375; doi:10.1186/s12966-018-0659-3)
Supplement: Supplementary file 2 — Changes in self-reported physical activity and sedentary time variables (baseline to 12-months) between HIGH and MODERATE groups in BETA, Alberta, Canada, 2010–2014. (DOCX 23 kb) [file 12966_2018_659_MOESM2_ESM.docx]

**Additional file 2.** Changes in self-reported physical activity and sedentary time variables (baseline to 12-months) between HIGH and MODERATE groups in BETA, Alberta, Canada, 2010-2014.

| **Outcome measure ^a^** | **Baseline**  M (SD) | **12-months**  M (SD) | **LS Mean Change ^b^**  M (95% CI) | *P* value ^c^ | **LS Group Difference ^b^**  M (95% CI) | *P* value ^d^ |
| --- | --- | --- | --- | --- | --- | --- |
| Total activity (MET-h/d)  HIGH  MODERATE    Total activity (MET-h/wk)  HIGH  MODERATE | 13.5 (6.2)  13.6 (6.7)  94.3 (43.4)  95.1 (46.6) | 17.3 (7.4)  16.1 (7.4)  121.4 (51.8)  112.8 (51.5) | 3.83 (2.87, 4.80)  2.57 (1.59, 3.55)  26.82 (20.06, 33.57)  17.97 (11.12, 24.82) | < 0.001  < 0.001 | 1.26 (-0.11, 2.64)  8.84 (-0.79, 18.48) | 0.07 |
| Occupational activity (MET-h/d)  HIGH  MODERATE  Occupational activity (MET-h/wk)  HIGH  MODERATE | 5.3 (4.9)  5.1 (5.0)  37.2 (34.3)  35.7 (35.1) | 5.6 (5.7)  5.6 (5.6)  39.5 (39.8)  39.2 (39.5) | 0.36 (-0.34, 1.06)  0.46 (-0.25, 1.16)  2.52 (-2.35, 7.39)  3.20 (-1.74, 8.14) | 0.31  0.20 | -0.10 (-1.09, 0.90)  -0.68 (-7.63, 6.28) | 0.85 |
| Household activity (MET-h/d)  HIGH  MODERATE  Household activity (MET-h/wk)  HIGH  MODERATE | 6.8 (4.7)  7.0 (4.6)  47.8 (32.6)  48.7 (32.3) | 6.6 (4.6)  7.0 (5.2)  46.0 (32.4)  49.3 (36.4) | -0.27 (-0.86, 0.32)  0.11 (-0.49, 0.71)  -1.89 (-6.00, 2.24)  0.78 (-3.40, 4.95) | 0.37  0.71 | -0.38 (-1.22, 0.46)  -2.66 (-8.54, 3.22) | 0.37 |
| Recreational activity (MET-h/d)  HIGH  MODERATE  Recreational activity (MET-h/wk)  HIGH  MODERATE | 1.2 (1.4)  1.4 (1.9)  8.5 (9.5)  9.8 (13.4) | 5.0 (2.9)  3.4 (2.1)  35.1 (20.6)  23.8 (14.6) | 3.74 (3.38, 4.10)  2.05 (1.69, 2.42)  26.18 (23.66, 28.70)  14.38 (11.83, 16.94) | < 0.001  < 0.001 | 1.69 (1.17, 2.20)  11.80 (8.20, 15.40) | < 0.001 |
| Transportation activity (MET-h/d)  HIGH  MODERATE    Transportation activity (MET-h/wk)  HIGH  MODERATE | 0.1 (0.3)  0.1 (0.3)  0.8 (1.9)  0.9 (2.1) | 0.1 (0.3)  0.1 (0.2)  0.7 (2.3)  0.6 (1.5) | -0.01 (-0.05, 0.03)  -0.04 (-0.08, -0.003)  -0.09 (-0.37, 0.18)  -0.30 (-0.57, -0.02) | 0.50  0.03 | 0.03 (-0.03, 0.08)  0.21 (-0.18, 0.59) | 0.30 |
| Total sedentary time (h/d)  HIGH  MODERATE  Total sedentary time (h/wk)  HIGH  MODERATE | 10.7 (3.6)  11.0 (3.4)  74.9 (25.2)  77.0 (23.8) | 10.2 (3.5)  10.5 (3.3)  71.4 (24.5)  73.5 (23.1) | -0.32 (-0.80, 0.16)  -0.13 (-0.63, 0.37)  -2.24 (-5.60, 1.12)  -0.91 (-4.41, 2.59) | 0.20  0.60 | -0.18 (-0.81, 0.44)  -1.26 (-5.67, 3.08) | 0.56 |
| Occupational sedentary time (h/d)  HIGH  MODERATE  Occupational sedentary time (h/wk)  HIGH  MODERATE | 2.2 (2.1)  2.1 (2.0)  15.4 (14.7)  14.7 (14.0) | 2.2 (2.0)  2.2 (2.1)  15.4 (14.0)  15.4 (14.7) | 0.23 (-0.02, 0.49)  0.34 (0.07, 0.60)  1.61 (-0.14, 3.43)  2.38 (0.49, 4.2) | 0.07  0.01 | -0.10 (-0.43, 0.22)  -0.7 (-3.01, 1.54) | 0.53 |
| Leisure sedentary time (h/d)  HIGH  MODERATE  Leisure sedentary time (h/wk)  HIGH  MODERATE | 8.5 (3.1)  8.9 (3.2)  59.5 (21.7)  62.3 (22.4) | 8.0 (3.0)  8.3 (2.9)  56.0 (21.0)  58.1 (20.3) | -0.55 (-0.98, -0.13)  -0.47 (-0.91, -0.03)  -3.85 (-6.86, -0.91)  -3.29 (-6.37, -0.21) | 0.01  0.04 | -0.08 (-0.63, 0.46)  -0.56 (-4.41, 3.22) | 0.76 |

**Note:** CI, confidence interval; d, day; h, hours; LS, least-squares; M, mean; MET, metabolic equivalent of task; SD, standard deviation; wk, week.

^a^ n = 180 and 175 for the HIGH and MODERATE groups, respectively, for the PYTPAQ; n = 166 and 159 for the HIGH and MODERATE groups, respectively, for the SIT-Q.

^b^ Least-square group mean of the High and Moderate exercise groups and their between-group differences were estimated from general linear model specified as: physical activity and sedentary time changes from baseline to 12-months = β0 + β1 (intervention group) + β2 (baseline outcome value) + β3 (age) + β4 (study site) + β5 (baseline BMI) + β6 (baseline VO2peak) + β7 (employment status for SIT-Q variables only).

^c^ *P* value for the test of significance for the null hypothesis that the LS mean difference across time equals 0.

^d^ *P* value for the test of significance for the null hypothesis that the LS mean difference between the two intervention groups equals 0.
