# Supplementary material for: Effects of prescribed aerobic exercise volume on physical activity and sedentary time in postmenopausal women: a randomized controlled trial
Source: Int J Behav Nutr Phys Act. 2018 Mar 21;15:27. doi: 10.1186/s12966-018-0659-3 (PMC5863375; doi:10.1186/s12966-018-0659-3)
Supplement: Supplementary file 3 — Changes in self-reported physical activity and sedentary time variables (12- to 24-months) between HIGH and MODERATE groups in BETA, Alberta, Canada, 2010–2014. (DOCX 22 kb) [file 12966_2018_659_MOESM3_ESM.docx]

**Additional file 3.** Changes in self-reported physical activity and sedentary time variables (12- to 24-months) between HIGH and MODERATE groups in BETA, Alberta, Canada, 2010-2014.

| **Outcome measure ^a^** | **12-months**  M (SD) | **24-months**  M (SD) | **LS Mean Change ^b^**  M (95% CI) | *P* value ^c^ | **LS Group Difference ^b^**  M (95% CI) | *P* value ^d^ |
| --- | --- | --- | --- | --- | --- | --- |
| Total activity (MET-h/d)  HIGH  MODERATE  Total activity (MET-h/wk)  HIGH  MODERATE | 17.6 (7.1)  16.4 (7.2)  123.0 (49.9)  115.1 (50.6) | 15.2 (8.1)  14.8 (6.9)  106.7 (56.4)  103.9 (48.3) | -2.13 (-3.12, -1.13)  -1.82 (-2.83, -0.80)  -14.88 (-21.85, -7.91)  -12.73 (-19.84, -5.63) | < .001  < .001 | -0.31 (-1.73, 1.12)  -2.15 (-12.14, 7.84) | 0.67 |
| Occupational activity (MET-h/d)  HIGH  MODERATE  Occupational activity (MET-h/wk)  HIGH  MODERATE | 5.6 (5.6)  5.6 (5.6)  39.2 (39.4)  38.9 (39.1) | 5.6 (5.6)  5.1 (5.3)  39.3 (39.2)  35.8 (36.9) | 0.02 (-0.62, 0.66)  -0.45 (-1.10, 0.20)  0.14 (-4.35, 4.62)  -3.15 (-7.72, 1.42) | 0.95  0.18 | 0.47 (-0.45, 1.39)  3.28 (-3.14, 9.70) | 0.32 |
| Household activity (MET-h/d)  HIGH  MODERATE  Household activity (MET-h/wk)  HIGH  MODERATE | 6.7 (4.6)  7.4 (5.4)  46.7 (32.3)  51.6 (37.5) | 6.8 (5.2)  7.0 (4.7)  47.9 (36.7)  48.8 (32.7) | -0.03 (-0.69, 0.64)  -0.20 (-0.87, 0.48)  -0.19 (-4.82, 4.45)  -1.39 (-6.11, 3.34) | 0.94  0.56 | 0.17 (-0.78, 1.12)  1.20 (-5.45, 7.85) | 0.72 |
| Recreational activity (MET-h/d)  HIGH  MODERATE  Recreational activity (MET-h/wk)  HIGH  MODERATE | 5.2 (2.8)  3.4 (2.1)  36.4 (19.9)  24.0 (14.5) | 2.6 (2.4)  2.6 (2.3)  18.2 (16.8)  18.3 (16.4) | -2.01 (-2.37, -1.66)  -1.42 (-1.79, -1.06)  -14.10 (-16.57, -11.62)  -9.97 (-12.50, -7.44) | < .001  < .001 | -0.59 (-1.11, -0.07)  -4.13 (-7.78, -0.48) | 0.03 |
| Transportation activity (MET-h/d)  HIGH  MODERATE    Transportation activity (MET-h/wk)  HIGH  MODERATE | 0.1 (0.3)  0.1 (0.2)  0.7 (1.9)  0.6 (1.5) | 0.2 (0.5)  0.1 (0.3)  1.3 (3.7)  1.0 (2.2) | 0.09 (0.03, 0.15)  0.05 (-0.01, 0.11)  0.63 (0.20, 1.05)  0.35 (-0.09, 0.79) | 0.004  0.12 | 0.04 (-0.05, 0.13)  0.28 (-0.34, 0.89) | 0.38 |
| Total sedentary time (h/d)  HIGH  MODERATE  Total sedentary time (h/wk)  HIGH  MODERATE | 9.9 (3.2)  10.5 (3.3)  69.3 (22.4)  73.5 (23.1) | 9.7 (3.3)  10.3 (3.5)  67.9 (23.1)  72.1 (24.5) | -0.16 (-0.65, 0.33)  0.11 (-0.41, 0.63)  -1.12 (-4.55, 2.31)  0.77 (-2.87, 4.41) | 0.52  0.67 | -0.27 (-0.92, 0.37)  -1.89 (-6.44, 2.59) | 0.41 |
| Occupational sedentary time (h/d)  HIGH  MODERATE  Occupational sedentary time (h/wk)  HIGH  MODERATE | 2.1 (2.0)  2.2 (2.1)  14.7 (14.0)  15.4 (14.7) | 2.0 (1.9)  2.1 (1.9)  14.0 (13.3)  14.7 (13.3) | -0.13 (-0.37, 0.12)  0.02 (-0.24, 0.29)  -0.91 (-2.59, 0.84)  0.14 (-1.68, 2.03) | 0.32  0.86 | -0.15 (-0.47, 0.17)  -1.05 (-3.29, 1.19) | 0.36 |
| Leisure sedentary time (h/d)  HIGH  MODERATE  Leisure sedentary time (h/wk)  HIGH  MODERATE | 7.8 (2.9)  8.2 (2.9)  54.6 (20.3)  57.4 (20.3) | 7.8 (3.1)  8.2 (3.2)  54.6 (21.7)  57.4 (22.4) | -0.01 (-0.45, 0.42)  0.08 (-0.37, 0.54)  -0.07 (-3.15, 2.94)  0.56 (-2.59, 3.78) | 0.96  0.71 | -0.09 (-0.66, 0.47)  -0.63 (-4.62, 3.29) | 0.74 |

**Note:** CI, confidence interval; d, day; h, hours; LS, least-squares; M, mean; MET, metabolic equivalent of task; SD, standard deviation; wk, week.

^a^ n = 160 and 154 for the HIGH and MODERATE groups, respectively, for the PYTPAQ; n = 152 and 144 for the HIGH and MODERATE groups, respectively, for the SIT-Q.

^b^ Least-square group mean of the High and Moderate exercise groups and their within- and between-group differences were estimated from general linear models specified as: physical activity and sedentary time changes from 12- to 24-months = β0 + β1 (intervention group) + β2 (12-month outcome value) + β3 (age) + β4 (study site) + β5 (baseline BMI) + β6 (baseline VO2peak) + β7 (employment status for SIT-Q variables only).

^c^ *P* value for the test of significance for the null hypothesis that the LS mean difference across time equals 0.

^d^ *P* value for the test of significance for the null hypothesis that the LS mean difference between the two intervention groups equals 0.
