# Supplementary material for: Effects of prescribed aerobic exercise volume on physical activity and sedentary time in postmenopausal women: a randomized controlled trial
Source: Int J Behav Nutr Phys Act. 2018 Mar 21;15:27. doi: 10.1186/s12966-018-0659-3 (PMC5863375; doi:10.1186/s12966-018-0659-3)
Supplement: Supplementary file 4 — Changes in self-reported physical activity and sedentary time variables (baseline to 24-months) between HIGH and MODERATE groups in BETA, Alberta, Canada, 2010–2014. (DOCX 23 kb) [file 12966_2018_659_MOESM4_ESM.docx]

**Additional file 4** Changes in self-reported physical activity and sedentary time variables (baseline to 24-months) between HIGH and MODERATE groups in BETA, Alberta, Canada, 2010-2014.

| **Outcome measure ^a^** | **Baseline**  M (SD) | **24-months**  M (SD) | **LS Mean Change ^b^**  M (95% CI) | *P* value ^c^ | **LS Group Difference ^b^**  M (95% CI) | *P* value ^d^ |
| --- | --- | --- | --- | --- | --- | --- |
| Total activity (MET-h/d)  HIGH  MODERATE  Total activity (MET-h/wk)  HIGH  MODERATE | 13.5 (6.1)  13.7 (6.7)  94.8 (42.5)  95.6 (47.0) | 15.2 (8.0)  14.9 (6.9)  106.1 (56.3)  104.4 (48.5) | 1.53 (0.50, 2.57)  1.33 (0.27, 2.39)  10.74 (3.48, 18.00)  9.30 (1.88, 16.72) | 0.004  0.014 | 0.21 (-1.28, 1.69)  1.44 (-8.97, 11.85) | 0.79 |
| Occupational activity (MET-h/d)  HIGH  MODERATE  Occupational activity (MET-h/wk)  HIGH  MODERATE | 5.3 (4.9)  5.1 (5.0)  37.0 (34.1)  35.6 (34.9) | 5.5 (5.6)  5.2 (5.3)  38.8 (39.1)  36.2 (37.1) | 0.31 (-0.38, 1.00)  0.05 (-0.65, 0.76)  2.15 (-2.68, 6.98)  0.36 (-4.57, 5.30) | 0.38  0.89 | 0.26 (-0.73, 1.25)  1.79 (-5.13, 8.72) | 0.61 |
| Household activity (MET-h/d)  HIGH  MODERATE  Household activity (MET-h/wk)  HIGH  MODERATE | 6.9 (4.7)  7.1 (4.8)  48.1 (32.7)  49.4 (33.3) | 6.9 (5.2)  7.0 (4.7)  48.0 (36.5)  48.9 (32.6) | -0.12 (-0.82, 0.58)  0.02 (-1.70, 0.73)  -0.86 (-5.75, 4.03)  0.12 (-4.88, 5.12) | 0.73  0.96 | -0.14 (-1.14, 0.86)  -0.98 (-8.00, 6.04) | 0.78 |
| Recreational activity (MET-h/d)  HIGH  MODERATE  Recreational activity (MET-h/wk)  HIGH  MODERATE | 1.3 (1.4)  1.4 (2.0)  8.9 (9.7)  9.7 (13.7) | 2.6 (2.4)  2.6 (2.3)  18.1 (16.8)  18.2 (16.4) | 1.25 (0.90, 1.60)  1.28 (0.93, 1.64)  8.77 (6.31, 11.23)  8.99 (6.48, 11.51) | < .001  < .001 | -0.03 (-0.54, 0.47)  -0.22 (-3.75, 3.31) | 0.90 |
| Transportation activity (MET-h/d)  HIGH  MODERATE  Transportation activity (MET-h/wk)  HIGH  MODERATE | 0.1 (0.3)  0.1 (0.3)  0.8 (1.9)  0.9 (2.1) | 0.2 (0.5)  0.1 (0.3)  1.3 (3.6)  1.0 (2.2) | 0.06 (0.0001, 0.13)  0.01 (-0.06, 0.07)  0.45 (0.001, 0.90)  0.06 (-0.40, 0.52) | 0.05  0.79 | 0.06 (-0.04, 0.15)  0.39 (-0.25, 1.03) | 0.24 |
| Total sedentary time (h/d)  HIGH  MODERATE  Total sedentary time (h/wk)  HIGH  MODERATE | 10.6 (3.6)  11.0 (3.4)  74.2 (25.2)  77.0 (23.8) | 9.7 (3.3)  10.3 (3.5)  67.9 (23.1)  72.1 (24.5) | -0.77 (-1.29, -0.25)  -0.35 (-0.89, 0.19)  -5.39 (-9.03, -1.75)  -2.45 (-6.23, 1.33) | 0.004  0.21 | -0.42 (-1.10, 0.26)  -2.94 (-7.70, 1.82) | 0.23 |
| Occupational sedentary time (h/d)  HIGH  MODERATE  Occupational sedentary time (h/wk)  HIGH  MODERATE | 2.1 (2.0)  2.1 (2.0)  14.7 (14.0)  14.7 (14.0) | 2.0 (1.9)  2.1 (1.9)  14.0 (13.3)  14.7 (13.3) | -0.03 (-0.29, 0.23)  0.17 (-0.10, 0.45)  -0.21 (-2.03, 1.61)  1.19 (-0.70, 3.15) | 0.83  0.21 | -0.21 (-0.54, 0.13)  -1.47 (-3.78, 0.91) | 0.24 |
| Leisure sedentary time (h/d)  HIGH  MODERATE  Leisure sedentary time (h/wk)  HIGH  MODERATE | 8.5 (3.2)  8.9 (3.2)  59.5 (22.4)  62.3 (22.4) | 7.8 (3.1)  8.2 (3.2)  54.6 (21.7)  57.4 (22.4) | -0.73 (-1.19, -0.26)  -0.53 (-1.01, -0.05)  -5.11 (-8.33, -1.82)  -3.71 (-7.07, -0.35) | 0.002  0.03 | -0.20 (-0.80, 0.40)  -1.4 (-5.6, 2.8) | 0.52 |

**Note:** CI, confidence interval; d, day; h, hours; LS, least-squares; M, mean; MET, metabolic equivalent of task; SD, standard deviation; wk, week.

^a^ n = 162 and 155 for the HIGH and MODERATE groups, respectively, for the PYTPAQ; n = 151 and 144 for the HIGH and MODERATE groups, respectively, for the SIT-Q.

^b^ Least-square group mean of the High and Moderate exercise groups and their within- and between-group differences were estimated from general linear models specified as: physical activity and sedentary time changes from baseline to 24-months = β0 + β1 (intervention group) + β2 (baseline outcome value) + β3 (age) + β4 (study site) + β5 (baseline BMI) + β6 (baseline VO2peak) + β7 (employment status for SIT-Q variables only).

^c^ *P* value for the test of significance for the null hypothesis that the LS mean difference across time equals 0.

^d^ *P* value for the test of significance for the null hypothesis that the LS mean difference between the two intervention groups equals 0.
